# Dying in self-defense: cell death signaling in animals and plants

**DOI:** 10.1038/s41418-023-01206-0

**Published:** 2023-08-10

**Authors:** Ana J. Garcia-Saez

**Affiliations:** Institute of Genetics, CECAD, University of Cologne, Cologne, Germany

**Keywords:** Cell biology, Biochemistry

The term “regulated cell death” encompasses a set of signaling pathways that have evolved to modulate the process of cellular demise in response to overwhelming stress or as part of a developmental program [1]. While certain primal cell death pathways have been identified in unicellular organisms as a form of altruism to stop infection spread in the community, it is in multicellular organisms that regulated cell death becomes central for life. Higher organisms have developed a variety of sophisticated and specialized pathways that trigger cell death in a controlled manner to fulfill a wide range of biological functions, ranging from the shaping of organs during embryo development, to the clearance of pathogen infection or the removal of transformed cells. The use of regulated cell death for these purposes is conserved in animals and plants. Yet, although the biochemical composition and organization of the signaling pathways may be diverse, the existence of common principles offers potential for synergy between the research fields to progress our knowledge about regulated cell death.

This special issue comprises a collection of ten reviews that explore the current understanding and latest developments in cell death signaling in plants and animals. Maekawa, Kashkar and Coll team up to provide a comparative perspective of immunogenic cell death in animals and plants [2]. They open the special issue by discussing the common strategies developed in both kingdoms of life to counteract pathogens attack and highlight the central role of alteration of the barrier function of the plasma membrane as a unifying mechanism. The comparative overview of the machineries involved suggests cross-seeding opportunities for the discovery of new mechanisms across kingdoms.

Proteases play a key role in the execution of many cell death processes in animals and plants by irreversible modifying the proteome of cells [3]. Van Breusegem and co-workers review the cellular mechanisms that control the activation and activity of proteases to regulate cell death, immune responses and development in plants [4]. They cover the cellular conditions that modulate the controlled activation of proteases in space and time and discuss the role of inhibitors, post-translational modifications and substrate expression in this process. Also at the level of proteases, Kietz and Meinander discuss the role of cysteine proteases, caspases, on the coordinated regulation of cell death and immune responses in *Drosophila melanogaster* [5]. The authors discuss the dual role of the seven caspases encoded in the fly genome, which goes beyond cell death signaling to immunoregulatory functions.

Several of the reviews continue on the immunological consequences of the activation of cell death pathways. Two of them explore the immunological aspects of mitochondrial apoptosis. Vingrer and Tait discuss the consequences of mitochondria-driven inflammation in the context of cell death [6]. They propose that the bacterial origin of mitochondria causes immune signaling responses similar to the bacterial activation of inflammation once the organelle contents spill to the cytosols as a consequence of mitochondrial permeabilization in apoptosis. The authors also comment on the therapeutic opportunities of exploiting mitochondria-dependent inflammation during cell death. Häcker and Haimovici focus instead on the induction of inflammatory signaling under conditions of sub-lethal mitochondrial permeabilization [7]. The authors review the evidence in favor of low levels of mitochondrial permeabilization happening naturally in the cell upon chemotherapeutic stress as well as during microbial infection. They defend the biological relevance of this process as a defense mechanism when cells come into contact with pathogens. Bock and Riley bring these concepts together and compare inflammatory signaling in the extrinsic and the intrinsic pathways of apoptosis [8]. They explore the circumstances under which physiological apoptosis is inflammatory and highlight cell death as a consequence of mitotic arrest as a special case for cell death-driven inflammation. Liccardi and Annibaldi connect cell death and inflammatory signaling in the context of necroptotic cell death instead [9]. They go beyond the TNF axis to discuss the physiological role of the pseudokinase MLKL in cellular defense against pathogens, and focus of the multiple levels of regulation of MLKL by post-translational modifications.

Because of the key role of regulated cell death in a variety of organismal processes, the dysregulation of cell death signaling is functionally linked to diseases ranging from cancer and neurodegeneration to autoimmune diseases. As a consequence, targeting cell death is a very promising target to improve human health [10]. With a focus at the level of the death receptors, Montinaro and Walczak evaluate the use of cell death to fight cancer. They focus on death receptor ligands, specifically TRAIL, as a tool to eliminate tumors by specifically killing cancer cells [11]. They discuss the main molecular mechanisms driving TRAIL resistance and the most promising strategies to overcome current limitations in their clinical application, especially in the context of combination therapies and optimization of TRAIL-receptor agonists. At the organismal level, Peltzer and coworkers discuss the connection between cell death and obesity [12]. They propose the idea that obesity is connected to low levels of chronic inflammation supported by TNF signaling and cell death within the adipose tissue. The authors also discuss the role of inflammation in organismal metabolism and the concept of metabolic inflammation. Finally, Yusupova and Fuchs explore the role of cell competition in mammalian stem cells [13]. They cover the biological settings in which cell competition is of biggest relevance, ranging from development and tissue maintenance to cancer formation, and discuss the mechanisms and cell death pathways involved in the resulting cell elimination.

Together, these reviews provide a broad perspective of the state of the art in cell death research and highlight the paramount role of regulating cell death in orchestrating immune responses at the tissue level. The classical concept of regulated cell death as a mechanism for controlled cellular removal is giving way to a more comprehensive picture in which dying cells communicate with the tissue microenvironment to maintain homeostasis and signal in case of damage [14].
